# *Bm*K-YA, an Enkephalin-Like Peptide in Scorpion Venom

**DOI:** 10.1371/journal.pone.0040417

**Published:** 2012-07-09

**Authors:** Yan Zhang, Junyan Xu, Zhiwei Wang, Xiuli Zhang, Xinmiao Liang, Olivier Civelli

**Affiliations:** 1 Department of Pharmacology, University of California Irvine, Irvine, California, United States of America; 2 Key Laboratory of Separation Science for Analytical Chemistry, Dalian Institute of Chemical Physics, Chinese Academy of Sciences, Dalian, China; 3 Department of Developmental and Cell Biology, University of California Irvine, Irvine, California, United States of America; 4 Department of Pharmaceutical Sciences, University of California Irvine, Irvine, California, United States of America; University of Rouen, France

## Abstract

By screening extracts of venom from the Asian scorpion *Buthus martensii* Karsch (*Bm*K) for their abilities to activate opioid receptors, we have identified *Bm*K-YA, an amidated peptide containing an enkephalin-like sequence. *Bm*K-YA is encoded by a precursor that displays a signal sequence and contains four copies of *Bm*K-YA sequences and four of His^4^-*Bm*K-YA, all flanked by single amino acid residues. *Bm*K-YA and His^4^-*Bm*K-YA are amidated and thus fulfill the characteristics expected of bioactive peptides. *Bm*K-YA can activate mammalian opioid receptors with selectivity for the δ subtype while His^4^-*Bm*K-YA is inactive at opioid receptors. The discovery of *Bm*K-YA suggests that scorpion venom may represent a novel source of bioactive molecules targeting G protein-coupled receptors (GPCRs) and reveal additional insights on the evolution of the opioid precursors.

## Introduction

Animal venoms constitute a vast library of biologically active peptides that are directed at a variety of membrane proteins. It is estimated that more than 10 million peptide toxins exist in 1400 species of scorpions, 400 species of snakes, 600 species of sea cone snails and 35000 species of spiders [1]. However, only a very small portion (less than 0.02%) of the estimated natural bank has been identified. It is becoming clear that venom peptides have diverse pharmacological properties and that several of these peptide toxins are useful as molecular tools for the study of their receptors [2], and may represent a unique source of leads and structure templates for the development of novel therapeutic molecules and insecticides [3]. Venoms can be used as the natural equivalents of large combinatorial libraries in drug discovery. Indeed a number of peptide toxins have been used *in vivo* for proof-of-concept studies, and several have undergone preclinical or clinical development for the treatment of pain, diabetes, multiple sclerosis and cardiovascular diseases [4]–[10]. Most venoms are known to contain peptide toxins that act on ion channels. Only a few are known to act on GPCRs. Indeed, among the 1800 toxins described in 2006 [11], less than 30 are known to be active on GPCRs [12].

The Asian scorpion *Bm*K is widely distributed in Mongolia, Korea and China where it has been used in Traditional Chinese Medicines (TCMs) for thousands of years as a source of pain relieving drugs. Animal study has shown that scorpion venom do not elicit dependence [13]. This suggests that further exploration of *Bm*K might provide a potential analgesic medicine without addictive properties. In recent decades, *Bm*K venom has been extensively studied and has led to the discovery of more than a hundred peptides through biochemical purification or deduced from gene cloning [14]–[21]. These peptides exhibit a wide range of physiological and pharmacological activities and have been developed as biopesticides, vaccines, cancer treatments, and protein engineering scaffolds [22], [23]. These scorpion peptides are mainly interacting with ion channels [15], but very little is known about their capacity to modulate GPCR activity.

In this study, we have used *Bm*K scorpion venom as a source of new ligands for opioid receptors. The opioid receptors are GPCRs that are activated by endogenously produced opioid peptides that contain an enkephalin sequence at their N-termini and also by exogenously administered opiates, such as morphine, a well known analgesic drug. Pharmacological and biochemical evidence supports the existence of three major subtypes of opioid receptors, μ, δ and κ [24]. Herein, we described the purification and biochemical characterization of a novel peptide, *Bm*K-YA that displays a sequence related to the enkephalin sequence and is encoded by a precursor that contains not only four sequences of *Bm*K-YA but also four of His^4^-*Bm*K-YA. *Bm*K-YA can activate mammalian opioid receptors. These data indicate that *Bm*K-YA and His^4^-*Bm*K-YA are bioactive peptides and reveal additional insights on the evolution of opioid precursors.

## Materials and Methods

### Material*s*

The crude venom of *Bm*K was collected by electrical stimulation of the telson of the scorpion and lyophilized in Luoyang city, Henan Province, China. HPLC-grade water was purified with a Milli-Q system (Millipore, Bedford, MA). Acetonitrile (ACN) was purchased from Fisher Scientific (Fair Lawn, NJ, USA). Trifluoroacetic acid (TFA) and ortho-phosphoric acid was from Tedia, USA. Formic acid was obtained from Acros (Geel, Belgium). Triethylamine was obtained from Sigma-Aldrich (St.Louis, Mo, USA). Fluo-4 AM was purchased from Molecular Probes (Eugene, OR). Lipofectamine was purchased from Invitrogen (Carlsbad, CA). All other reagents were analytical grade and used without purification.

### Venom pre-treatment and Purification procedures

Solid phase extraction (SPE) cartridges packed with octadecyl sorbent (30 g sorbent/cartridge) were washed with methanol, 60% ACN/0.1% TFA and 5% ACN/0.1% TFA sequentially. The lyophilized crude venom (ca. 4g) was dissolved in 5% ACN/0.1% TFA aqueous solution and loaded onto the cartridges in batches. Each cartridge was rinsed with 100 mL 5% ACN/0.1% TFA aqueous solution to remove some polar substances, and then the peptides were eluted with 100 mL 60%ACN/0.1%TFA aqueous solution. In total, 1.3 L eluate was collected, pooled and lyophilized by the Refrigerated CentriVap Centrifugal Concentrator (Labconco, Kansas, USA). Finally, about 1.2 g lyophilized sample was generated.

*Bm*K-YA was purified by an offline two-dimensional chromatographic strategy,in which reverse phase liquid chromatography (RPLC) was combined with hydrophilic interaction chromatography (HILIC). Briefly, the SPE treated venom was redissolved in 5% ACN/0.05% TFA aqueous solution and fractionated by a preparative C18 column (XTerra MS C18,100×19 mm i.d., 5 µm particle size, 120 Å pore size, Waters). The sample was loaded on the column at 100 mg per run. The mobile phase was composed of 0.05% (v/v) TFA aqueous solution (mobile phase A) and ACN with 0.05% (v/v) TFA (mobile phase B). The gradient was run from 5% to 35% mobile phase B over 50 min. The flow rate was 17 mL/min and the elution was monitored by MS (Micromass ZQ2000). The passive splitter was about 1/3000. Mass Scans were acquired in positive ion mode from m/z 300–2000.

Fractions were collected automatically at 1 minute interval and denoted as Fraction 1 to Fraction 50. The fraction with the same elution time from each round of HPLC was pooled and evaporated to dryness in the Centrifugal Concentrator.

The fraction of interest (Fraction 17) was further purified on a homemade Click Maltose column (150×4.6 mm, 5 µm). This stationary phase was prepared through click chemistry as described earlier [25]. The mobile phase was composed of water (A), ACN (B) and 100 mM triethylamine phosphate (TEAP) buffer (pH 2.3) (C). The gradient was from 10%A/85%B/5%C to 45% A/50%B/5%C in 40 min, then from 45% A/50%B/5%C to 50% A/45%B/5%C in 20 min. The absorbance was measured at 214 nm.

### Peptide de novo sequencing and synthesis

ESI MS and ESI MS/MS were performed using a nano-LC-MS/MS system (nano-Acquity™ coupled to a Q-TOF premier™, Waters, Manchester, UK). Scans were acquired in positive ion mode from m/z 500–1500 Da for MS analysis and m/z 50–1000 Da for MS/MS analysis with nanospray voltage at 2.0kV. The source temperature was maintained at 80°C and the cone voltage was set to 35 kV. The collision energy for MS/MS analysis of *Bm*K-YA was set at 27 eV. The amino acid sequence of *Bm*K-YA was analyzed by MassLynx 4.1 software incorporating the MaxEnt3 deconvolution algorithm and PepSeq tools (Waters, Manchester, UK).

The *Bm*K-YA peptide was synthesized through solid-phase synthesis (ChinaPeptides Co., Ltd, Shanghai, China) on an Applied Biosystems 433A system as described previously [26]. Briefly, the peptide was synthesized in a small reactor, on the 2-chlorotrityl chloride resin preloaded with Fmoc-L-Ala-OH. Side chain protecting groups used for tri-functional residues were tert-butyl for Tyr and trityl (Trt) for Asn. After full assembly was completed, the resin was treated with TFA/water/ethanedithiol/thioanisol (94%/2.5%/2.5%/1%) for 120 min. The crude peptide was extracted with cold diethyl ether six times and dried under a flow of nitrogen. Finally, the extracted peptide was purified to homogeneity through a Kromasil 100-5 C18 column (250×4.6 mm, 5 µm) with a linear gradient from 5% to 35% ACN/0.1%TFA in 30 min.

### Plasmid construction and stable cell lines

All GPCRs used in this study were amplified from human cDNA library (Clontech, Palo Alto, CA) and cloned into pcDNA 3.1 (-) (Invitrogen, Carlsbad, CA). The sequences were confirmed by sequencing from both ends and with internal primers by Laragen (Los Angeles, CA). Human embryonic kidney-293 T cells (HEK293T) were cultured in Dulbecco’s Minimum Essential Medium (DMEM) supplemented with 10% fetal calf serum (FBS). The stable cell lines expressing human opioid receptors μ, δ or κ individually were created as previously reported [27]. The individual human opioid receptors μ, δ or κ DNA plasmid were cotransfected with Gqαi3, a chimeric G protein with which opioid receptors can be redirected to mediate intracellular calcium mobilization upon stimulation. Transfection was carried out with lipofectamine using the protocol provided by the supplier. Stable cell clones were selected in the presence of 200 µg/mL G418, 200 µg/mL hygromycin and 200 µg/mL zeocin.

### Identification of a cDNA clone encoding *Bm*K-YA

Total RNA was extracted from Asian scorpion *Bm*K using Trizol Reagent (Invitrogen, CA). Messenger RNA was purified with Qiagen Oligotex mRNA Kit. Based on the information obtained from direct peptide sequencing, RACE-ready cDNA and subsequent amplification of 5′ and 3′ ends were performed using Smart RACE cDNA kit from Clontech. RACE primers are:5′-GTTTTCACCGCTTTAATTTATCTACATAGAATG-3′and 5′-CATTCTATGT- AGATAAATTAAAGCGGTGAAAAC-3′. PCR products were cloned into pcDNA3.1/V5-His-TOPO vector and sequenced by Laragen (Los Angelus, CA).

### Ca^2+^ response monitored by Fluorometric Imaging Plate Reader Assay (FLIPR)

The assay was performed as reported earlier [28]. Briefly, the stable cells were seeded into poly-D-lysine-coated black wall, clear-bottom 96-well plates at a density of 80,000 cells per well. Twenty-four hours later the medium was removed and replaced with 100 µL of dye loading solution (2 µM Fluo-4 AM dissolved in FLIPR buffer, which consists of 0.2 mg/mL pluronic acid in 1×Hank’s buffer supplemented with 20 mM HEPES, pH 7.4) for 1 h at 37°C. The cells were then washed 3 times with FLIPR buffer prior being assayed. The samples, which were re-dissolved in dimethyl sulphoxide (DMSO) and stored in 96-well drug plates, were diluted with FLIPR buffer and then added into the cells within 4 sec automatically. The intracellular Ca^2+^ concentration was monitored at 520 nm with excitation wavelength at 488 nm over a period of 4 min.

### Data processing

EC_50_ values and curve fitting were determined using Graphpad Prism (GraphPad Software, Inc., San Diego, CA). The maximal stimulation was determined by the response of the selective ligands endomorphin-1, deltrophin, and dynorphin A in µ-, δ- and κ-expressing stable cell lines, respectively. Data from each dose response curve were normalized to the maximal stimulation of each cell line. Potency is determined by EC_50_. Efficacy is determined by Emax, which is the ratio of the maximal response of each peptides and the maximal stimulation induced by selective ligands in corresponding opioid receptor-expressing cells. When the Emax reaches 100%, the peptide is considered a full agonist.

## Results

### Identification and characterization of a novel enkephalin-like peptide from venom

**Figure 1:**
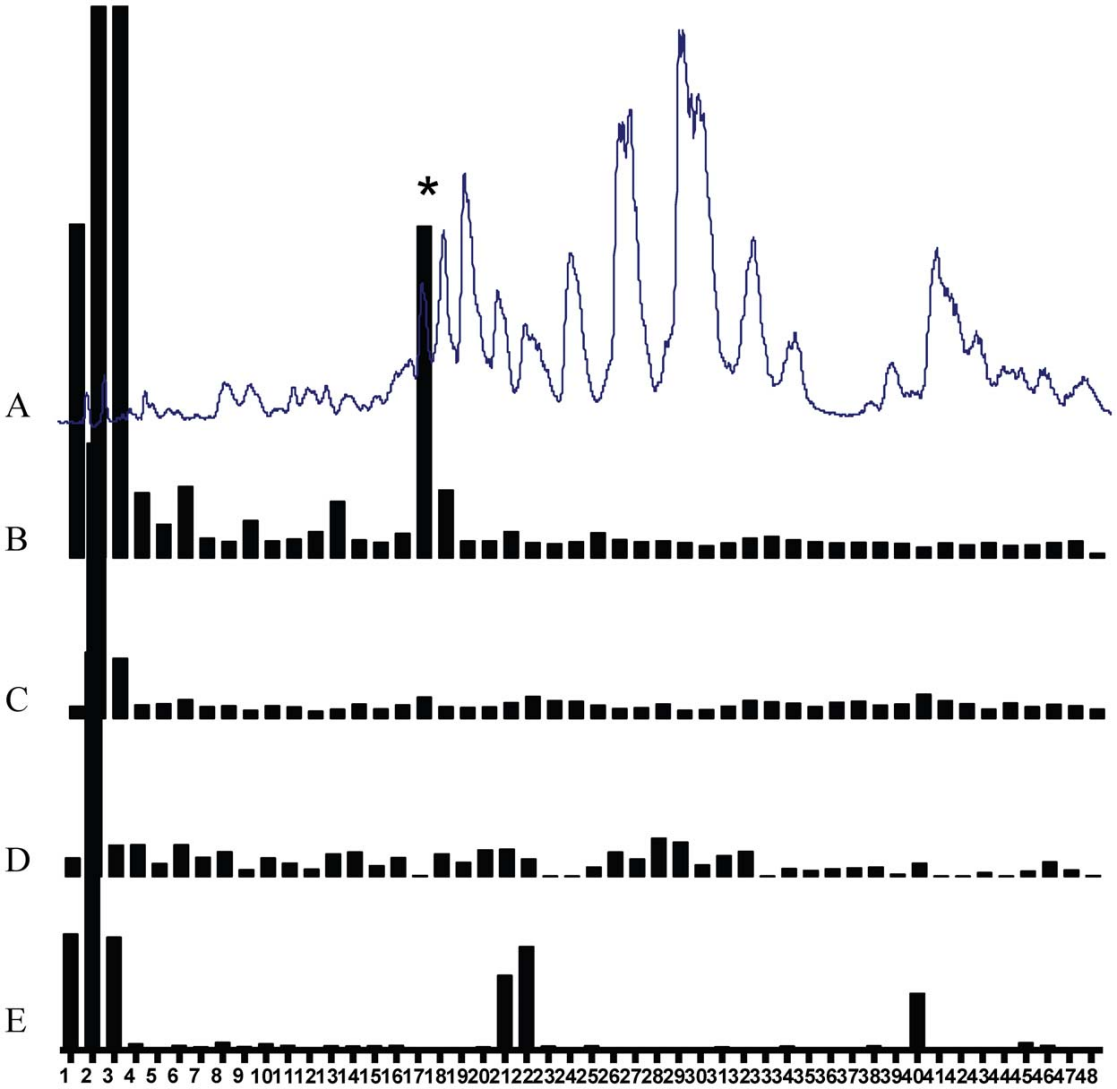
Fractionation of scorpion venom peptides and their opioid activities. (A) Scorpion venom was fractionated on a prep C18 column using a preparative HPLC system. One hundred milligrams of peptide samples were loaded per run. Fractions were eluted with a 50 min linear gradient from 5% B (ACN with 0.05% (v/v) TFA) to 35% B at a flow rate of 17 mL/min. The elution was monitored by MS. The activities of the fractions were detected in (B) δ-expressing cells. (C) μ-expressing cells. (D) κ-expressing cells. (E) vector-expressing cells (negative control).

**Figure 2:**
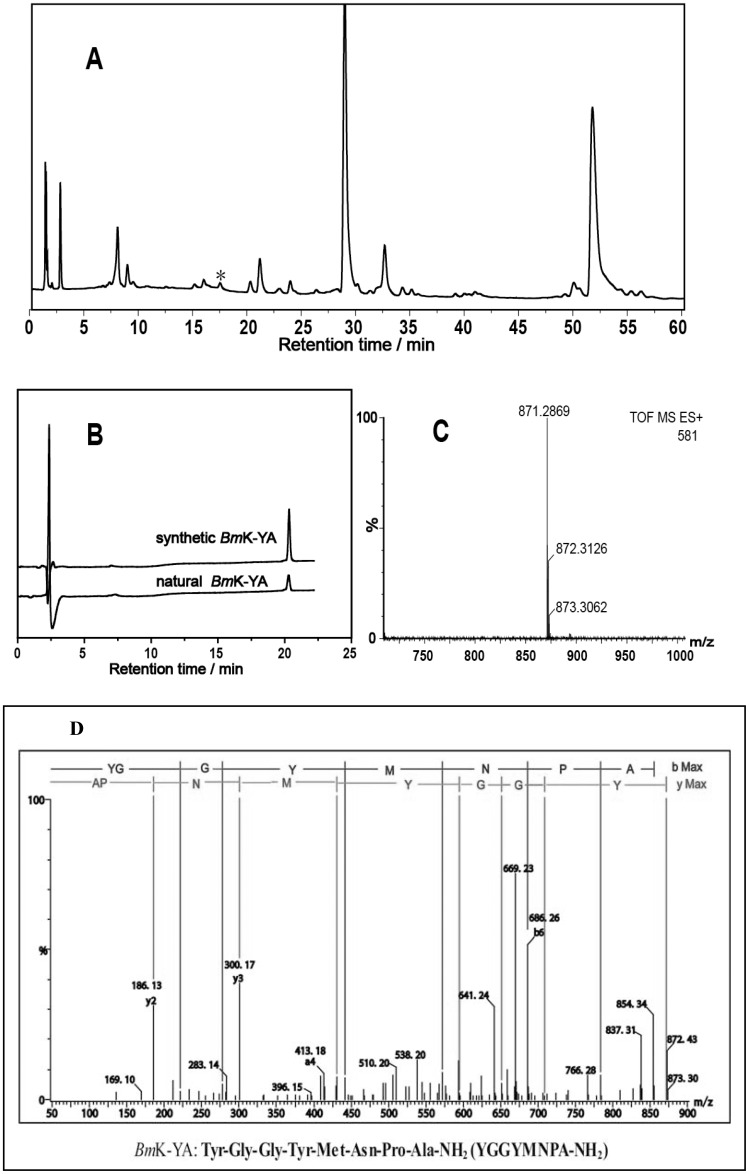
Purification of *Bm*K-YA from Fraction 17. (A) Chromatographic profile of F17 separated by Click Maltose column using HILIC mode. The mobile phase was composed of water (A), ACN (B) and 100 mM TEAP buffer (pH 2.3) (C). The gradient was from 10% A to 45% A over 40 min, then from 45% A to 50% A over 20 min under a constant 5% C. (B) RPLC analysis and comparison of the purified natural *Bm*K-YA and the synthetic one. The mobile phase B (ACN with 0.1%TFA) was from 5% to 35% in 25 min on a Xterra MS C18 column at a flow rate of 0.2 mL/min. Absorbance was measured at 220 nm. (C) Q-TOF mass spectra of the natural *Bm*K-YA with a [M+H]^+^ monoisotopic mass of 871.3088. (D) Single charged and deconvoluted (MaxEnt3 processed spectra) CID spectra and amino acid sequence of *Bm*K-YA.

Scorpion venom was fractionated by a preparative C18 column into 50 fractions. These fractions were screened against three individual opioid receptors-expressing cell lines and one vector-expressing HEK293T cell line. Intracellular Ca^2+^ changes were monitored using the FLIPR system. A reproducible and robust change in Ca^2+^ concentration in δ-expressing cells but not in vector-expressing cells was observed in Fraction 17 (labeled with asterisk in Fig. 1). Fraction 17 was further purified in an analytical scale Click Maltose column (Fig. 2A, B), yielding the component of interest (Fig. 2A, peak labeled with asterisk). HPLC and Mass spectrometry analysis of this peak revealed a single peptide with 870.3 atomic mass unit (Fig 2B, C). The amino acid sequence Tyr-Gly-Gly-Tyr-Met-Asn-Pro-Ala-NH_2_ (YGGYMNPA) was obtained by nanoLC-Q-TOF-MS/MS and PepSeq software (Fig. 2 D). Included in the sequence is a C-terminal amidation, a typical post-translation in scorpion venom. We have termed this peptide *Bm*K-YA, based on the genus of the scorpion and its first and last amino acid. By searching NCBI data bank it is revealed that *Bm*K-YA is a fragment of a large predicted protein (accession number: AAD39510).

### Chemical synthesis of *Bm*K-YA

*Bm*K-YA was synthesized chemically. The synthesized material was found to have the same retention time as the native peptide (Fig. 2B) and the same monoisotopic mass [M+H] (MW 871.3032). The synthesized peptide was tested in δ-expressing cells and shown to exhibit a reproducible δ receptor agonist response indicating that the synthesized peptide is the same as the native one found in scorpion venom. Because of this, the synthesized peptide was used for further pharmacological characterization.

### cDNA Cloning and sequencing

**Figure 3:**
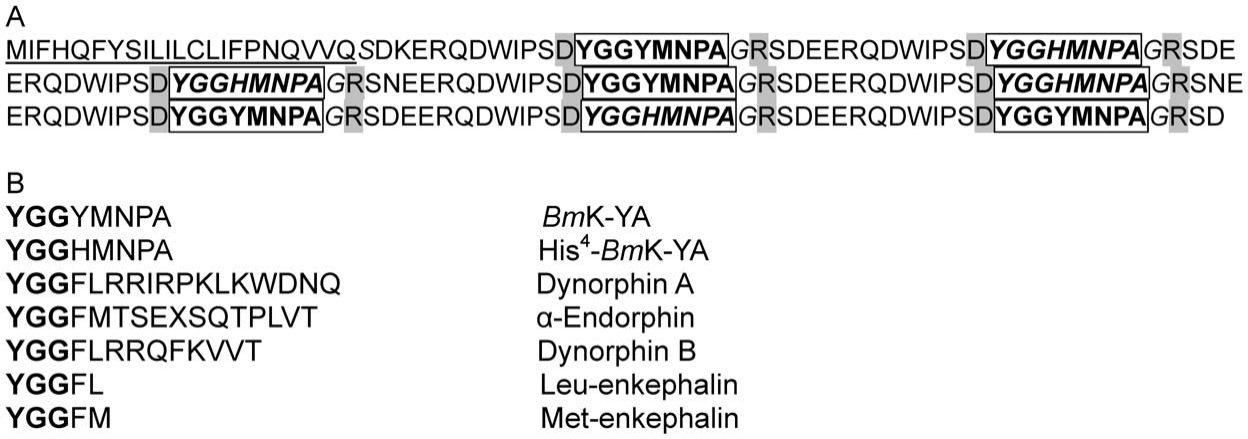
(A) Amino acid sequence of the *Bm*K-YA precursor. The signal peptide is underlined. The amino acid in grey indicates cleavage sites (R, D). The glycine residues expected to serve as amide donors are shown in italic. The sequences of *Bm*K-YA and His^4^-*Bm*K-YA (italic) are framed in bold. **(B)** Comparison of *Bm*K-YA and His^4^-*Bm*K-YA to the opioid peptides. Identical amino acid residues are shown in bold.

Degenerate oligonucleotides synthesized according to the amino acid sequence of *Bm*K-YA were used to screen a cDNA library. This procedure allowed the identification of a long DNA segment corresponding to the majority of the *Bm*K-YA gene. The full DNA sequence was obtained using the nucleotides indicated in *Material and Methods*. Fig. 3A shows that ther *Bm*K-YA cDNA precursor encodes a 200-residue protein containing a 23-residue signal peptide, followed by 177-residues. Aspartic acid cleavage at C-terminal has been reported in murine [29], [30]. The single arginine cleavage found at N-terminal of the *Bm*K-YA occurs in a number of other mammalian neuropeptide presursors. The cDNA encoding the mature peptides contained 4 copies of the repeated sequence “YGGYMNPA”, the *Bm*K-YA, and 4 copies of the sequence “YGGHMNPA”, predicted to be a novel peptide named His^4^-*Bm*K-YA. The glycine residues at the C-terminals of *Bm*K-YA or His^4^-*Bm*K-YA are expected to be coverted into amide groups. The sequences of the cDNA and the purified peptide demonstrate that *Bm*K-YA is a natural peptide in scorpion.

### Pharmacological characterization of *Bm*K-YA

**Figure 4:**
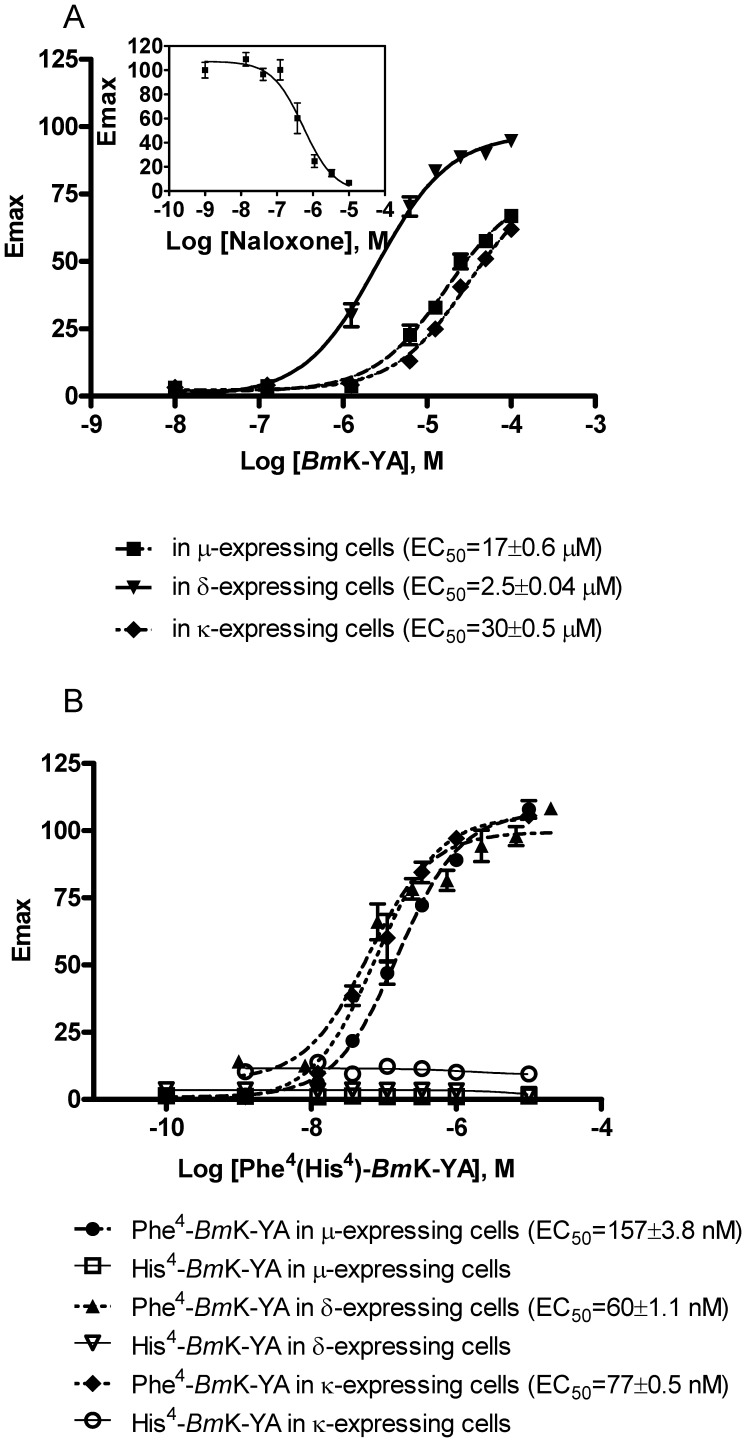
*Bm*K-YA and related peptides activity at opioid receptors. (A) Dose response curves of intracellular Ca^2+^ mobilization induced by *Bm*K-YA in opioid receptors-expressing HEK293T cells. The EC_50_ was 17±0.6 μM, 2.5±0.04 μM and 30±0.5 μM (mean±SE, n = 3) in μ-, δ- and κ-expressing HEK293T cells, respectively. The insert shows the inhibitory effect of naloxone on *Bm*K-YA-induced Ca^2+^ release in δ-expressing cells (IC_50_: 539±13 nM (mean±SE, n = 3)); *Bm*K-YA concentration of used in this experiment was 2.5 μM. (B) Dose response curves of intracellular Ca^2+^ mobilization induced by Phe^4^-*Bm*K-YA and His^4^-*Bm*K-YA in μ-, δ- and κ-expressing HEK293T cells, respectively. The EC_50_ of Phe^4^-*Bm*K-YA was 157±3.8 nM, 60±1.1 nM, and 77±0.5 nM (mean±SE, n = 3) in μ-, δ- and κ-expressing HEK293T cells, respectively.

*Bm*K-YA acted as a full agonist when tested in δ-expressing cells as shown in Fig. 4A. No response was observed in vector-expressing cells (data not shown). *Bm*K-YA has a similar efficacy but exhibited lower potency (2.5 µM) when compared with deltorphin, a selective δ opioid receptor agonist, (potency 3.1 nM [27]). We also tested the effects of *Bm*K-YA on µ- and κ-expressing cells. As shown in Fig. 4A, at concentrations up to 100 µM, *Bm*K-YA does not reach a maximal effect and exhibit lower potencies (approximately17 µM and 30 µM, respectively).

The δ-activity of *Bm*K-YA was inhibited by naloxone, in a dose-dependent manner (Fig. 4A, insert) (IC_50_ 539 nM), providing additional evidence that *Bm*K-YA can interact with opioid receptors.

### Synthesis and activities of His^4^-*Bm*K-YA and Phe^4^-*Bm*K-YA

His^4^-*Bm*K-YA was synthesized and tested for its ability to activate opioid receptors. As shown in Fig. 4B, no activity was detected in the concentration range from 1 nM to 50 µM.

According to the typical opioid peptide sequence, Phe^4^-*Bm*K-YA was synthesized in which phenylalanine (F) was substituted for tyrosine in the fourth position. As shown in Fig. 4B, this substitution dramatically increased the activity of the peptide which then has potencies of 157 nM, 60 nM and 77 nM, in μ-, δ-, and κ-expressing cells, respectively.

## Discussion

The toxins found in venomous animals have been optimized over time to aid in prey capture and digestion and also to help the animals defend themselves. While venomous animals receive their fair share of notoriety for the painful (and often deadly) effects of their bites and stings, their venoms have been harnessed for the treatment of human diseases for thousands of years. In recent years, venoms have been subjected to more rigorous scientific investigation as a potential source of new therapeutic entities. Currently, five venom-derived peptide drugs are on the market, and many more are in pre-clinical or clinical development for indications such as cancer, pain, heart disease, stroke, and diabetes [31]. Animal venoms represent a valuable source of untested bioactive molecules, as the venoms of only a few hundred species have been studied to date.

Animal venom peptides that are active at GPCRs can be divided into two families [12]. The members of the first family mimic the natural agonist at the target receptor. Peptides belonging to this family are snake sarafotoxins, which are functional analogs of the endogenous endothelins [32], the cone snail toxin conopressin, which is similar to the arginine-vasopressin peptide [33], and the cone snail toxin contulakin-G, which is similar to the neurotensin peptide [34]. The second family of GPCR toxins consists of highly reticulated peptides with folds unrelated to those of natural ligands [1], [35]–[40]. We have discovered *Bm*K-YA, the first scorpion venom peptide that displays a primary structure resembling that of the enkephalin-like peptides. *Bm*K-YA thus belongs to the family of venom peptides that mimic the natural agonists and suggests that scorpion venom may represent a novel source of GPCRs ligands.

By analyzing the sequence of the protein encoding *BmK-*YA, we found a polyprotein containing four *Bm*K-YA (YGGYMNPA) and four His^4^-*Bm*K-YA (YGGHMNPA). This polyprotein contains a typical signal sequence, which indicates that it is secreted. Furthermore *Bm*K-YA and His^4^-*Bm*K-YA can be amidated suggesting that they are bioactive. The organization of this precursor is reminiscent to that of the mammalian opioid peptide precursors where multiple sequence-related peptides within a single genomic transcript. Excluding the endomorphins, the classical opioid peptides are derived from three larger precursors: proopiomelanocortin (POMC), proenkephalin (PENK) and prodynorphin (PDYN), which encodes for one, seven and three enkephalin-containing sequences (YGGFM or YGGFL). Compared to these, the precursor encoding *Bm*K-YA, contains eight copies of enkephalin-like sequences (YGGYM or YGGHM). Interestingly the core *Bm*K-YA enkephalin-like sequences are followed by four conserved residues (NPAG), of which the glycine residue serves as amide donor. This amidation is thought to be mediated by a specific amidation enzyme [41], [42]. Indeed, two amidated enkephalin-like peptides, amidorphin and metorphamide, have been reported in mammals [43], [44]. A data base search did not yield any significant hits in mammalian genomes although eight repeats of the sequence (…RGGYVNPAG…) are found as part of the TBC1 domain family member 14 protein. This is however a non secreted protein.

We show that *Bm*K-YA *in vitro* interacts with the three subtypes of opioid receptors, μ, δ and κ, but with preference to the δ subtype. Its selectivity to the δ-subtype is 6.8 times higher than that to the μ, and 12 times higher than that to the κ subtype. It therefore displays a pharmacological profile that is different from morphine. *Bm*K-YA is a full agonist at the δ receptor with an EC_50_ of 2.5 µM while morphine is only a partial agonist with an EC_50_ of 15 µM [27]. Although both molecules can activate δ receptors with low potency, morphine cannot stimulate δ receptors as effectively as *Bm*K-YA at high concentrations. On the other hand, morphine is a full agonist at the μ receptor with an EC_50_ of 180 nM [27], while *Bm*K-YA is only a partial agonist with an EC_50_ of 17 µM. Thus *Bm*K-YA might induce fewer of the side effects associated with μ receptors. This may serve as a starting point for structure-function relationship studies leading to design specific antinociceptive drugs.

Whether *Bm*K-YA acts at opioid receptors in the scorpion is not known but not expected. The fact that *Bm*K-YA is encoded in a precursor that also contains His^4^-*Bm*K-YA leads us to hypothesize that both peptides should act at the same receptors. However, His^4^-*Bm*K-YA is inactive at the opiod receptors. Indeed, it is the specific His^4^ substitution that is responsible for the lack of activity since Phe^4^-*Bm*K-YA (YGGFMNPA), which contains a copy of Met-enkephalin, exhibits high affinity for the opioid receptors.

Several lines of evidence indicate that *Bm*K-YA is the first member of a new bioactive peptide family in scorpions. First, *Bm*K-YA is encoded by a precursor that can be secreted. Second, the organization of this precursor is similar to that of the mammalian opioid peptide precursors with multiple sequence-related peptides within a single genomic transcript. Third, *Bm*K-YA and His^4^-*Bm*K-YA are flanked by processing cleavage sites and can be amidated. Fourth, the NH2- tripeptide YGG sequence of *Bm*K-YA and His^4^-*Bm*K-YA is identical to the core sequence YGGF of the opioid peptides (Fig. 3B) and thus suggest evolutionary conservation. Whether they act as bioactive peptides *in vivo* will however await the identification of their receptor(s).

The discovery of *Bm*K-YA and its identification as an enkephalin-like peptide demonstrates that relatively “primitive” organisms may possess opioid-like systems. The present study supports previous work that have characterized opioid peptides (enkephalin-containing) in invertebrate, for example, the mussel *Mytilus edulis* [45] and the digestive system of the scallop *Chalmys farreri* [46]. It has also been reported on the basis of binding and immunocytochemical analyses that δ opioid receptors subtypes may exist in invertebrates [47], [48]. *Bm*K-YA is the first invertebrate peptide that displays a similar but not identical enkephalin sequence. Because enkephalin sequences are found in invertebrates and vertebrates while the *Bm*K sequence is not, it is reasonable to assume that enkephalins served as templates for *Bm*K-YA. Since the *Bm*K-YA gene is not found in other species by database bank search it may be unique to the scorpion. The final appearance of *Bm*K-YA gene may be the result of a genome duplication [49] which started with PENK. Over time one copy of a duplicated PENK gene may retain the PENK organization scheme and the function of the ancestral gene, while the other copy would accumulate amino acid substitution and diverge into a unique member of the *Bm*K-YA gene family. Because we did not, in our purification, identify other enkephalin-containing peptides such as true opioid peptides (N terminus:YGGF), we expect that they do not exist in scorpion. Thus *Bm*K-YA might have evolved to carry role(s) distinct from classical opioid function. This is reinforced by the coexistence of His^4^-*Bm*K-YA, which does not exhibit activity at mammalian opioid receptors. Also, the presence of these peptides in the venom of the scorpion is counterintuitive to them displaying an analgesic activity. Consequently we propose that these peptides must interact with receptors that are divergent of the mammalian opioid receptors and that, in the venom, *Bm*K-YA and His^4^-*Bm*K-YA may have evolved for specialized use, such as prey capture, defense or immune response.
